# Independent endpoint event review for the elimination of reporting bias in an open label phase III pharmaceutical trial

**DOI:** 10.1186/1745-6215-16-S2-P188

**Published:** 2015-11-16

**Authors:** Peter Hall, Anna Waterhouse, Isabelle Smith, Julia Brown, Walter Gregory, Guenther Steger, Richard Bell, David Cameron

**Affiliations:** 1 University of Edinburgh, Edinburgh, UK; 2 University of Leeds, Leeds, UK; 3 Andrew Love Cancer Centre, Victoria, Australia; 4 Medical University of Vienna, Vienna, Austria

## Background

Open label randomised controlled trials may be subject to bias where outcome ascertainment relies on treating clinician decisions. In the setting of a multinational randomised controlled open label phase three pharmaceutical trial we implemented a rigorous verification algorithm to mitigate against such bias. We report the impact of the algorithm's application on the primary endpoint.

## Method

The BEATRICE trial recruited 2591 patients with early surgically treated triple negative breast cancer from 360 sites in 37 countries into a two way randomisation. The primary endpoint was investigator reported invasive disease free survival (IDFS). A novel rigorous 103-step endpoint review algorithm was developed to provide central ratification in support of, but not mandating locally reported events.

## Results

IDFS events were reported in 393 patients. There was no statistically significant difference between the arms. The HR was 0•88 [95% CI 0•72-1•07] prior to algorithm application compared with 0.87 [95% CI 0.72-1.07]. 393 events, the algorithm input changed dates in 74 (19%), type of event in 7 (2%) and site of event in 11 (3%). The event number did not change.

## Conclusion

Reassuringly the primary results of this open label RCT did not change with enhanced independent interrogation and review of reported endpoints. Current standard trial reporting procedures appear to be adequate even in the open label setting.

